# Shared genetic investigation of asthma and blood eosinophils in relation to chronic rhinosinusitis

**DOI:** 10.1186/s13223-025-00956-5

**Published:** 2025-03-17

**Authors:** Xian Li, Jingyun Li, Siyao Xue, Yunbo Gao, Lianqi Wan, Chengshuo Wang, Yuan Zhang, Luo Zhang

**Affiliations:** 1 Department of Allergy, Beijing Tongren Hospital, Capital Medical University, No. 1, Dongjiaominxiang, DongCheng District, Beijing, 100730 P.R. China; 2 Department of Otolaryngology Head and Neck Surgery, Beijing Tongren Hospital, Capital Medical University, Beijing, 100730 China; 3 Beijing Key Laboratory of Nasal Diseases, Beijing Institute of Otolaryngology, Beijing, 100005 China; 4 Beijing Laboratory of Allergic Diseases and Beijing Key Laboratory of Nasal Diseases, Beijing Institute of Otolaryngology, Beijing, 100005 China; 5 Beijing Institute of Otolaryngology, No. 17, HouGouHuTong, DongCheng District, Beijing, 100005 P.R. China

**Keywords:** Asthma, Blood eosinophil, Chronic rhinosinusitis, Mendelian randomization

## Abstract

**Background:**

An epidemiological association among asthma, blood eosinophil level and chronic rhinosinusitis (CRS) is well established, but whether consistent genetic relationships exist, and whether this reflects a shared genetic etiology between CRS and asthma or blood eosinophil level remains unclear.

**Methods:**

Data from CRS patients (*N* = 1,255) and healthy controls (*N* = 1,032) were reviewed retrospectively to investigate associations between clinical characteristics and CRS. Data from white blood cells in the UK biobank (*N* = 173,480), asthma in the Trans-National Asthma Genetic Consortium (127,669) and CRS (*N* = 272,922) or nasal polyps (*N* = 264,107) in the FinnGen consortium were used to conduct genetic study, including linkage disequilibrium score regression analysis to detect genetic associations between aforementioned variables, Mendelian randomization (MR) analysis to investigate causal relationships of asthma and blood eosinophil levels on CRS, and Bayesian co-localization to consolidate MR findings and to identify shared genetic signals.

**Results:**

We found that blood eosinophil count, blood eosinophil percentages and asthma shared positive and causal genetic correlations with CRS (all q < 0.0001) and CRS with nasal polyps (CRSwNP) (all q < 0.0001) in both our observational and genetic study. Through colocalization analysis, 4 loci are shared among asthma, CRS and CRSwNP, 7 loci are shared among blood eosinophil count, CRS and CRSwNP, 2 loci are unique to blood eosinophil count and CRS, and 3 loci are unique to blood eosinophil count and CRSwNP.

**Conclusions:**

These findings contribute to understanding CRS etiology, and provide insights for intervention and treatment target for CRS comorbid with asthma or high blood eosinophil levels.

**Supplementary Information:**

The online version contains supplementary material available at 10.1186/s13223-025-00956-5.

## Background

Chronic rhinosinusitis (CRS) is a common disease of the upper airways that is characterized by a persistent inflammation in the mucosa of nose and paranasal sinuses. The persistent inflammation can cause perennial nasal blockage, rhinorrhea, and anterior/posterior nasal drip, which adversely influences quality of life due to headache, facial pain, and hyposmia [1]. Recently, CRS with nasal polyps (CRSwNP), one phenotype of CRS, has been getting more attention since its heterogenous and refractory intrinsic property [2]. The disease severity and inadequate control of CRSwNP is linked to asthma comorbidity and high blood eosinophil levels [3]. Meanwhile, both CRSwNP and asthma share prominent type 2 inflammation, which is characterized by the presence of extensive eosinophilic inflammation associated with type 2-related cytokines [4].

Previous genetic analysis found positive correlation between the effect of asthma-associated variants on the risk of asthma and CRSwNP (*R* = 0.61) [5]. Large-scale genome-wide association studies (GWAS) have identified over 150 independent loci associated with asthma and 10 loci with nasal polyps (e.g. *HLA-DQA1* and *IL33*), respectively [5–10]. Genetic studies have also determined the overlapping susceptibility loci of CRS and blood eosinophil count using data from European populations [11]. However, for most variations, causality is not determined, and the etiological effect of blood eosinophils on CRS is still disputed [12, 13], so the shared genetic loci among CRS, asthma, and blood eosinophil level need to be further clarified.

An important issue for clinicians in rhinology and respiratory clinics is how to treat patients with both CRS and asthma. Although numerous treatments are available for CRSwNP patients, high recurrence rate in CRSwNP patients with asthma comorbidity or high blood eosinophil levels are still not resolved completely [1]. Recently, biologicals (e.g. dupilumab) that have been used to treat CRSwNP with asthma can both improve upper and lower airway outcome measures [14]. However, hypereosinophilia and eosinophil-related pulmonary complications have also been reported following initiation of dupilumab [15, 16]. In this regard, an improved understanding of genetic relations among CRS, asthma and blood eosinophil count would lead to safer and more effective interventions for patients with both diseases individually and when they occur together.

In this study, we conducted observational and genetic study, including linkage disequilibrium score regression (LDSC) analysis, to detect relationships among asthma, blood eosinophil levels and CRS. Then we used single-variable Mendelian randomization (SVMR) and multivariable MR (MVMR) analysis to clarify causal relationships between asthma or blood eosinophils and CRS. Finally, colocalization analysis were used to identify shared variations between two traits. These results may provide a better understanding of CRS etiology and an insight of therapy strategies that can simultaneously address multiple comorbidities in CRS in the future.

## Methods

**Fig. 1:**
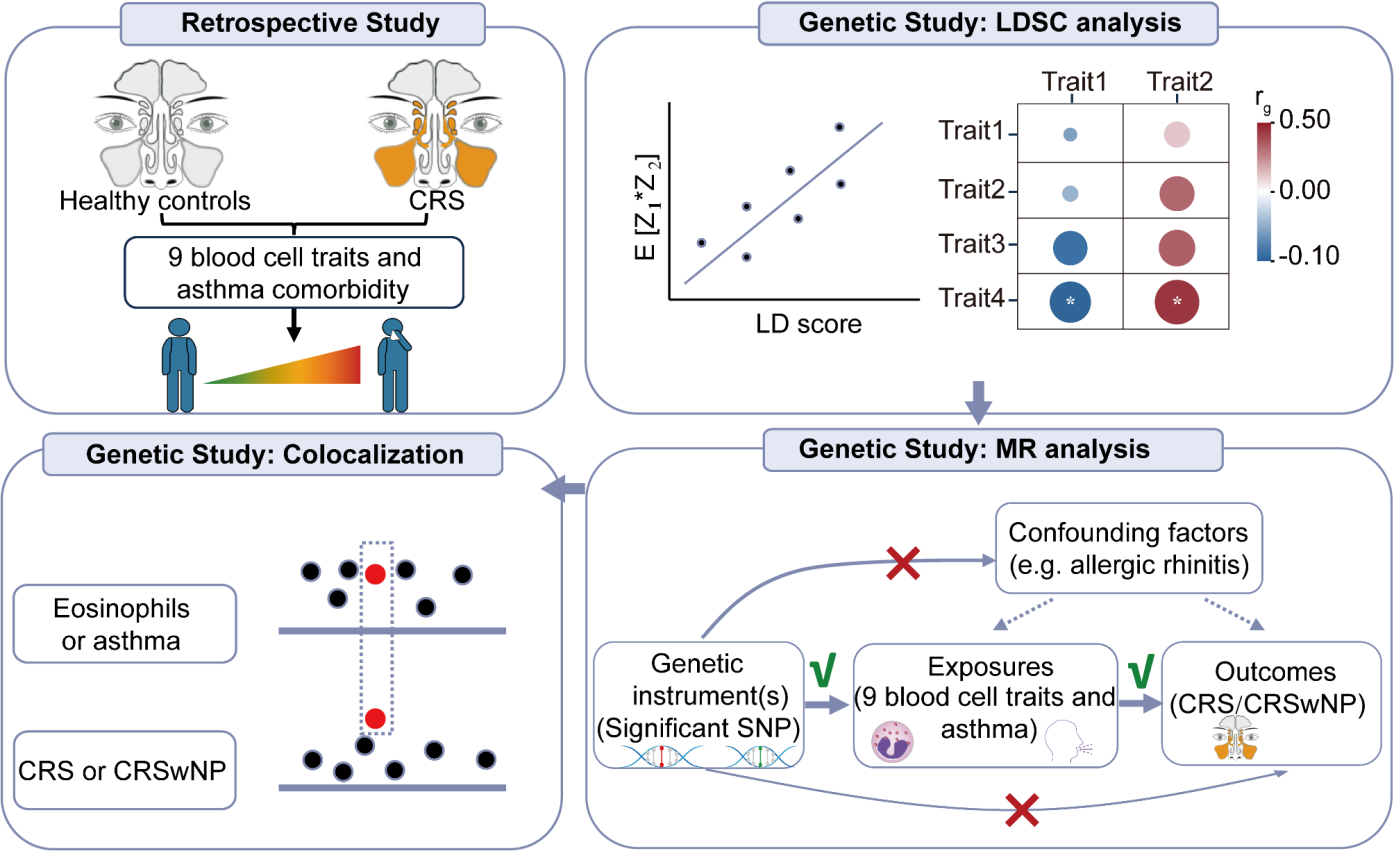
Overview of this study. First, we identified the profiles of blood cell traits and asthma comorbidity in healthy controls and CRS patients. Second, we adopted genetic study including LDSC analysis to clarify the genetic correlation of paired traits, single variable and multivariable MR to estimate the causal role of blood cell traits or asthma on CRS and CRSwNP and colocalization analysis to detect the shared causal genetic signals between traits. Abbreviations: CRS, chronic rhinosinusitis; CRSwNP, CRS with nasal polyps, LDSC: linkage disequilibrium score regression; MR, Mendelian randomization; SNP, single nucleotide polymorphism

Figure 1 shows the overall study design. Further details of the methods are provided as follows.

### Retrospective study

#### Participants and study design

Data were collected from CRS patients who underwent endoscopic sinus surgery at Beijing Tongren Hospital from September 2014 to August 2018. The patients had been diagnosed with CRS according to the European Position Paper on Rhinosinusitis and Nasal Polyps 2012 guidelines [17]. All CRS patients were adults with appropriate body mass index (BMI), routine blood tests, and serum total IgE. We excluded patients with choanalpolyps, cystic fibrosis, fungal sinusitis, or primary ciliary dyskinesia. Similarly, the control group data were collected from healthy adult individuals who had regular physical examinations at Beijing Tongren hospital physical examination center during the same period. None of these participants had any nasal and pulmonary symptoms and did not present with purulent secretion, edema/mucosal obstruction. This study was approved by the Ethics Committee of Beijing Tongren Hospital and written informed consent from each patient was obtained before data were collected.

#### Collection of clinical data and analysis

The weight and height of each participant was recorded during physical examination for the control group or after hospitalization for the CRS group. The BMI scores were determined using each person’s weight in kilogram and dividing it by the square of their height in meters (kg/m^2^). The recommended BMI cut-off values for Chinese people are as follows: 18.5–23.9 kg/m^2^ is considered normal, 24.0–27.9 is overweight, and ≥ 28 kg/m^2^ is obese [18]. Blood samples were taken during physical examination or before surgery, and analyzed by an automated analyzer to get the differential peripheral blood cell count. For CRS patients, comorbidity with asthma was also recorded. Evidence of nasal polyps was obtained using endoscopy. The serum total IgE (kU/I) was analyzed using an Immuno-Cap Phadiatop (Pharmacia, Uppsala, Sweden).

Observational data were analyzed using the SPSS version 23.0 software (IBM Corp, Armonk, NY). The Kolmogorov–Smirnov test was used to evaluate the normality of the data, and the non-normally distributed continuous variables were expressed as median and interquartile ranges. The Mann–Whitney U test was used when comparing two different groups. Categorical variables were expressed as numbers and percentages. Chi-square test was used to compare proportional differences between groups. Odds ratio (OR) and 95% confidence intervals (CI) were calculated using the logistic regression model. The Benjamini–Hochberg false discovery rate (q value) was used to adjust for multiple comparisons, and q < 0.05 was considered significant.

### Genetic study

The datasets used in this genetic study part and instrumental variable selection in mendelian randomization are detailed in Additional file 1.

#### LDSC regression

LDSC analysis regressed the chi-squared statistics for a single trait to estimate single nucleotide polymorphism (SNP)-based heritability (h^2^) or two traits to assess genetic correlation (r_g_). This analysis was conducted using the LDSC software based on GWAS summary statistics. Pre-estimated LD scores from the 1000 Genomes European reference population was used as reference [19]. Default settings were used in our analyses. q < 0.05 was considered significant.

#### Mendelian randomization

For MR analysis, we used inverse-variance weighted (IVW) MR as the primary method, which makes the fundamental assumption that all included SNPs are valid instrumental variables. Additional sensitivity analyses include MR-Egger and weighted median. MR-Egger regression can provide a valid effect estimate even though all SNPs are invalid instruments [20]. The weight median approach selects the median MR estimates as the causal estimate and provides a consistent effect estimate if more than 50% of the information comes from valid SNPs [21]. Cochrane’s Q value was used to assess the heterogeneity. The MR-Egger intercept and MR pleiotropy residual sum and outlier tests were used to detect horizontal pleiotropy [22, 23]. Leave-one-out analyses were also performed to detect high influence points [22]. The SVMR analysis was performed using TwoSampleMR (version 0.5.6) and MR (version 0.5.1) packages and the MVMR analysis was conducted using the MVMR packages (version 0.5.6) in R software 4.1.2 (https://www.r-project.org/). q < 0.05 was considered significant.

#### Colocalization analysis

We used Coloc, a Bayesian test to identify the probability of evidence consistent with a shared causal signal involved in blood eosinophil count and asthma with CRS and CRSwNP, respectively [24]. For these analyses, we considered the 70 SNPs that were significantly associated with blood eosinophil count or 13 SNPs associated with asthma were used as relevant instrumental variables. We extracted summary statistics for these variants (as well as variants 500 kb upstream and downstream [25]) from the blood eosinophil count or asthma GWAS and CRS or CRSwNP GWAS statistics. Bayesian colocalization was then implemented for each independent region in the R package coloc (https://CRAN.R-project.org/package=coloc). Briefly, Coloc estimate the prior probabilities for a SNP being associated with each trait (p1 and p2) and for a SNP being associated with both traits (p12) [24]. We used the default setting in our analyses. We set the threshold for strong evidence of colocalization at posterior probability ≥ 0.70 [26].

## Results

### Retrospective study

#### Blood eosinophil, monocyte levels and asthma are independent risk factors for CRSwNP

A total of 1,032 healthy participants, 1255 CRS patients, and 960 CRSwNP patients were included in this study. Detailed descriptive statistics for each subgroup are shown Table 1. Briefly, when compared to healthy controls, participants in the CRS and CRSwNP group had lower female proportions, higher white cells count, lower lymphocyte count and neutrophil percentage, higher blood monocyte and eosinophil levels (all q < 0.05). In addition, CRSwNP group had elder participants when compared to CRS group (q < 0.05). While the BMI index, overweight ratio, blood lymphocyte and neutrophil count did not show significant difference among three groups.

**Table 1:** Demographic and clinical characteristics of participants in healthy control, CRS, and CRSwNP groups

Variables	HC	CRS	CRSwNP	CRS vs. HC		CRSwNP vs. HC	
	(*N* = 1032)	(*N* = 1255)	(*N* = 960)	*P*	q	*P*	q
Age (years)	41 (33, 49)	42 (32, 53)	43 (33, 53)	0.25	0.33	**0.006**	**0.008**
Female, No. (%)	443 (42.90)	420 (33.50)	321(33.40)	**< 0.001**	**< 0.001**	**< 0.001**	**0.009**
BMI (kg/m^2^)	24.44 (22.14, 26.93)	24.68 (22.34, 27.00)	24.61 (22.34, 27.00)	0.86	0.93	0.57	0.16
BMI ≥ 24, No. (%)	574 (55.60)	719 (58.30)	562 (58.50)	0.45	0.53	0.17	0.44
Comorbid asthma (%)	-	188 (15.0)	179 (18.6)	-	-	-	-
Serum total IgE (kU/I)	-	73.00 (29.40–173.00)	77.80 (31.50–186.00)	-	-	-	-
Blood white cells count (×10^9^/L)	6.01 (5.11, 7.14)	6.35 (5.42, 7.42)	6.43 (5.45, 7.50)	**< 0.001**	**< 0.001**	**< 0.001**	**< 0.001**
Blood lymphocytes count (×10^9^/L)	1.99 (1.62, 2.41)	2.05 (1.67, 2.45)	2.06 (1.69, 2.48)	0.11	0.16	0.02	0.44
Blood lymphocytes percent (%)	33.40 (28.43, 38.60)	32.50 (27.20, 37.80)	32.25 (27.40, 37.40)	**0.002**	**0.003**	**0.001**	**0.002**
Blood neutrophils count (×10^9^/L)	3.52 (2.85, 4.36)	3.51 (2.80, 4.41)	3.57 (2.82, 4.42)	0.95	0.95	0.52	0.31
Blood neutrophils percent (%)	59.00 (53.60, 64.10)	56.10 (50.20, 62.30)	55.90 (50.20, 62.10)	**< 0.001**	**0.009**	**< 0.001**	**0.009**
Blood monocyte count (×10^9^/L)	0.25 (0.19, 0.34)	0.36 (0.28, 0.46)	0.36 (0.29, 0.46)	**< 0.001**	**< 0.001**	**< 0.001**	**< 0.001**
Blood monocyte percent (%)	4.08 (3.27, 5.46)	5.70 (4.50, 7.00)	5.70 (4.70, 6.90)	**< 0.001**	**< 0.001**	**< 0.001**	**< 0.001**
Blood eosinophils count (×10^9^/L)	0.10 (0.06, 0.18)	0.25 (0.12, 0.41)	0.27 (0.14, 0.44)	**< 0.001**	**< 0.001**	**< 0.001**	**< 0.001**
Blood eosinophils percent (%)	1.70 (1.06, 2.80)	3.80 (1.98, 6.20)	4.20 (2.30, 6.50)	**< 0.001**	**< 0.001**	**< 0.001**	**< 0.001**

Values are presented as the median (interquartile range) or numbers (percentage). q values are the P values adjusted for false discovery rate. Abbreviations: HC, healthy controls; CRS, chronic rhinosinusitis; CRSwNP, chronic rhinosinusitis with nasal polyps; BMI, body mass index

**Fig. 2:**
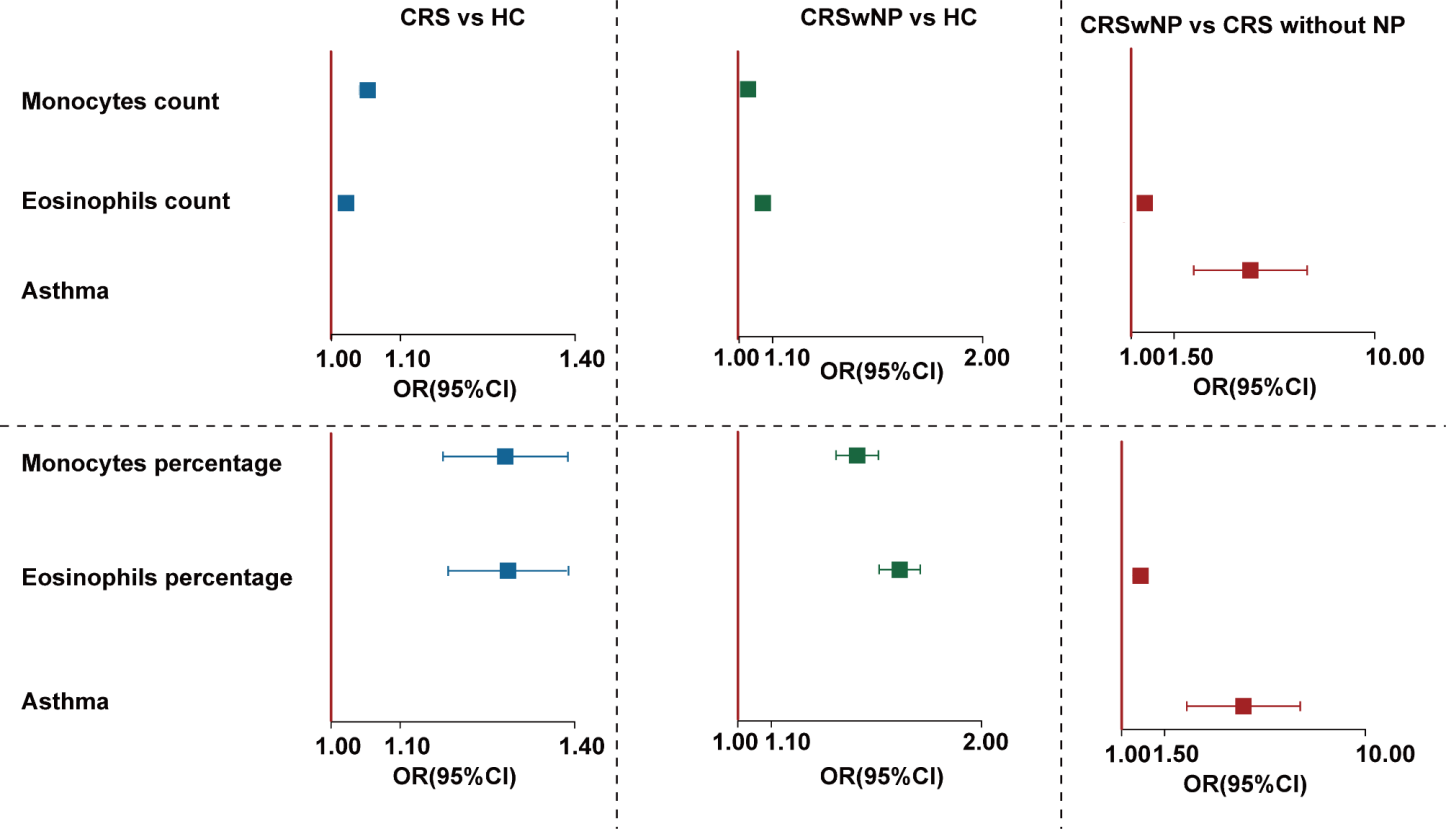
Logistic regression analysis of the risk factors associated with CRS. Results are expressed as the OR and 95% CI. Abbreviations: CRS, chronic rhinosinusitis; CRSwNP, chronic rhinosinusitis with nasal polyps; HC, healthy controls; OR, odds ratio; CI, confidence interval

Logistic regression analysis showed that blood monocyte count (OR = 1.05, 95%CI = 1.04–1.06, q < 0.001) and percentage (OR = 1.30, 95%CI = 1.21–1.40, q < 0.001), blood eosinophil count (OR = 1.02, 95%CI = 1.02–1.03, q < 0.001) and percentage (OR = 1.31, 95%CI = 1.22–1.40, q < 0.001) were risk factors for CRS patients when compared to healthy controls. And blood monocyte count (OR = 1.02, 95%CI = 1.01–1.02, q < 0.001) and percentage (OR = 1.35, 95%CI = 1.18–1.51, q < 0.001), blood eosinophil count (OR = 1.03, 95%CI = 1.02–1.03, q < 0.001) and percentage (OR = 1.62, 95%CI = 1.52–1.72, q < 0.001), and asthma comorbidity (OR = 3.80, 95%CI = 2.23–6.47, q < 0.001) were independent risk factors for CRSwNP when compared to healthy controls or CRS without nasal polyps (Fig. 2).

### Genetic study

#### Blood eosinophil levels and asthma are genetically related with CRSwNP

**Fig. 3:**
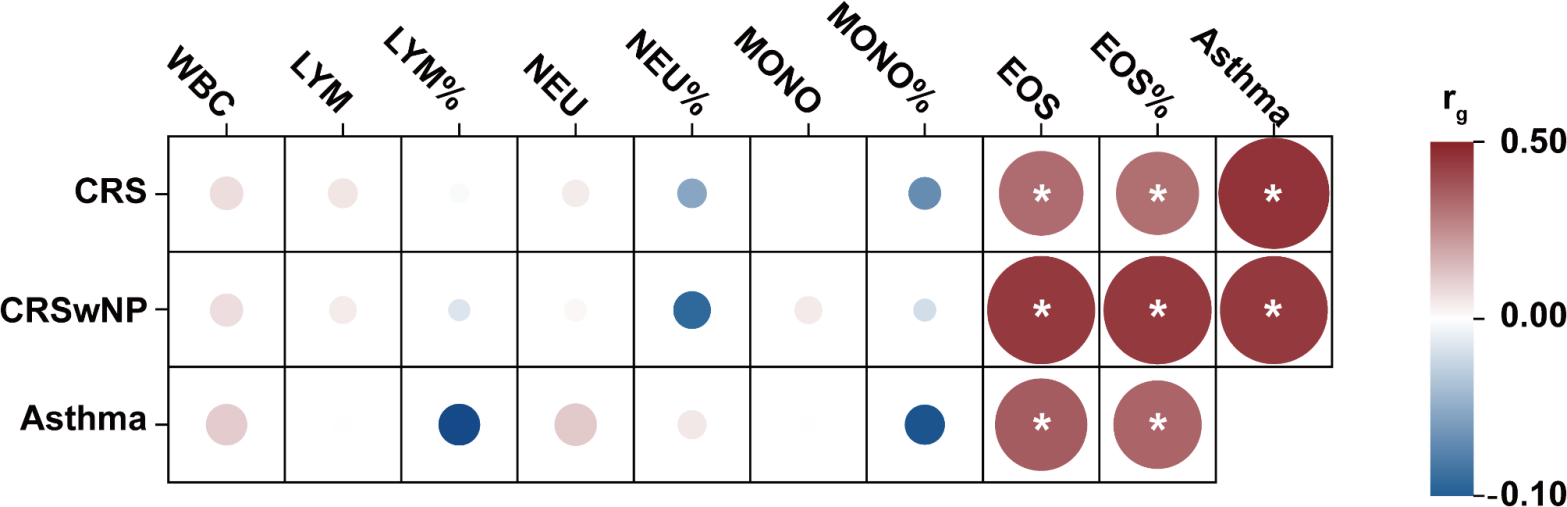
Heatmap of genetic correlations among blood cell traits and asthma on CRS, or CRSwNP. Asterisks indicate genetic correlations surviving multiple testing correction (q < 0.05). Detailed results after Benjamini–Hochberg correction are provided in Table S1 in Additional file 2. Abbreviations: CRS, chronic rhinosinusitis; CRSwNP, chronic rhinosinusitis with nasal polyps; WBC, white blood cell counts; LYM, blood lymphocyte counts; NEU, blood neutrophil count; MONO, blood monocyte count; EOS, blood eosinophil count

Then we performed LDSC regression analysis to assessed genetic correlations among blood cell traits, asthma and CRS or CRSwNP, respectively. As shown in Fig. 3, blood eosinophil counts and percentage showed significant, moderate, and positive genetic correlations with CRS (count: r_g_=0.33; percentages: r_g_=0.32, q < 0.001), CRSwNP (count: r_g_=0.45; percentages: r_g_=0.45; q < 0.001) and asthma (count: r_g_=0.36; percentages: r_g_=0.35; q < 0.001). Meanwhile, asthma was also genetically correlated with CRS (r_g_=0.46, q < 0.001) and CRSwNP (r_g_=0.45, q < 0.001). Detailed information was listed in Table S1 in Additional file 2.

#### Blood eosinophil and asthma have causal roles in CRSwNP

**Fig. 4:**
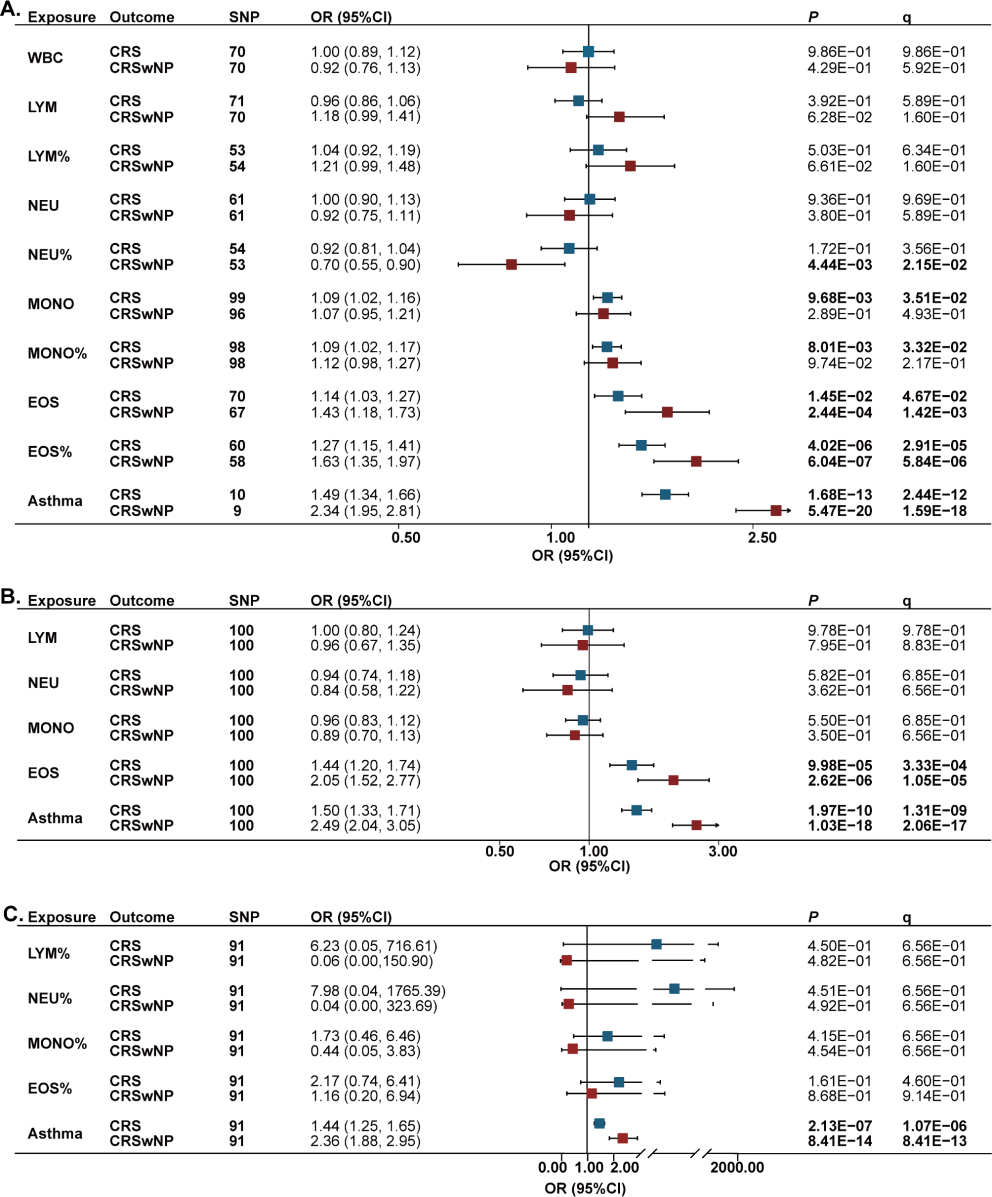
Single-variable (**A**) and multivariable MR analysis (**B** and **C**) to estimate the effect of white blood cell traits and asthma on CRS and CRSwNP. Associations were assessed using the inverse-variance weighted approach. Abbreviations: MR, Mendelian randomization; SNP, number of single nucleotide polymorphism; CRS, chronic rhinosinusitis; CRSwNP, chronic rhinosinusitis with nasal polyps; WBC, white blood cell counts; LYM, blood lymphocyte counts; NEU, blood neutrophil count; MONO, blood monocyte count; EOS, blood eosinophil count. q indicates the *P* values adjusted for the false discovery rate. q and *P* values < 0.05 are shown in bold

We further clarified the true causality among blood eosinophil level, asthma and other immune cell types associated with CRS and CRSwNP using SVMR analysis. The primary results are presented in Fig. 4A. After adjustments, in CRS patients, blood monocyte count (OR = 1.09, 95%CI = 1.02–1.16, q = 3.51 × 10^− 2^), blood monocyte percentage (OR = 1.09, 95%CI = 1.02–1.17, q = 3.32 × 10^− 2^), blood eosinophil count (OR = 1.14, 95%CI = 1.03–1.27, q = 4.67 × 10^− 2^), blood eosinophil percentage (OR = 1.27, 95%CI = 1.15–1.41, q = 2.91 × 10^− 5^), and asthma (OR = 1.49, 95%CI = 1.34–1.66, q = 2.44 × 10^− 12^) had positive causal effect on CRS (Fig. 4A, Table S2 in Additional file 2). In CRSwNP patients, genetically predicted blood neutrophil percentage (OR = 0.70, 95%CI = 0.55–0.90, q = 2.15 × 10^− 2^) had negative causal effect on CRSwNP, while blood eosinophil count (OR = 1.43, 95%CI = 1.18–1.73, q = 1.42 × 10^− 3^), blood eosinophil percentage (OR = 1.63, 95%CI = 1.35–1.97, q = 5.84 × 10^− 6^), and asthma (OR = 2.34, 95%CI = 1.95–2.81, q = 1.59 × 10^− 18^) had positive causal effect on CRSwNP (Fig. 4A, Table S3 in Additional file 2). The conventional IVW leave-one-out analysis did not identify any high leverage points with high influence, and the MR-Egger intercept analysis did not indicate horizontal pleiotropy (Table S2-S3 in Additional file 2). However, any potential influence of white blood cell traits on asthma was not detected (Table S4 in Additional file 2).

MVMR analysis was performed to correct confounding effects and to identify independent causal risk factors associated with CRS and CRSwNP, by adjusting for variables in absolute count and percentages of circulating blood white cells, respectively (Fig. 4B and C). The causal effect of blood eosinophil count on CRS (OR = 1.44, 95%CI = 1.20–1.74, q = 3.33 × 10^− 4^) and CRSwNP (OR = 2.05, 95%CI = 1.52–2.77, q = 1.05 × 10^− 5^) was still robust. Meanwhile, the causal effect of asthma on CRS (OR = 1.50, 95%CI = 1.33–1.71, q = 1.31 × 10^− 9^) and CRSwNP (OR = 2.49, 95%CI = 2.04–3.05, q = 2.06 × 10^− 17^) was also consistent with the effect estimated by SVMR. (Table S5-S6 in Additional file 2). MVMR-Egger sensitivity analysis showed consistent estimate effect. MR-Egger intercept analysis did not indicate horizontal pleiotropy (Table S5-S6 in Additional file 2).

#### Blood eosinophil and asthma share genetic signals with CRSwNP

**Fig. 5:**
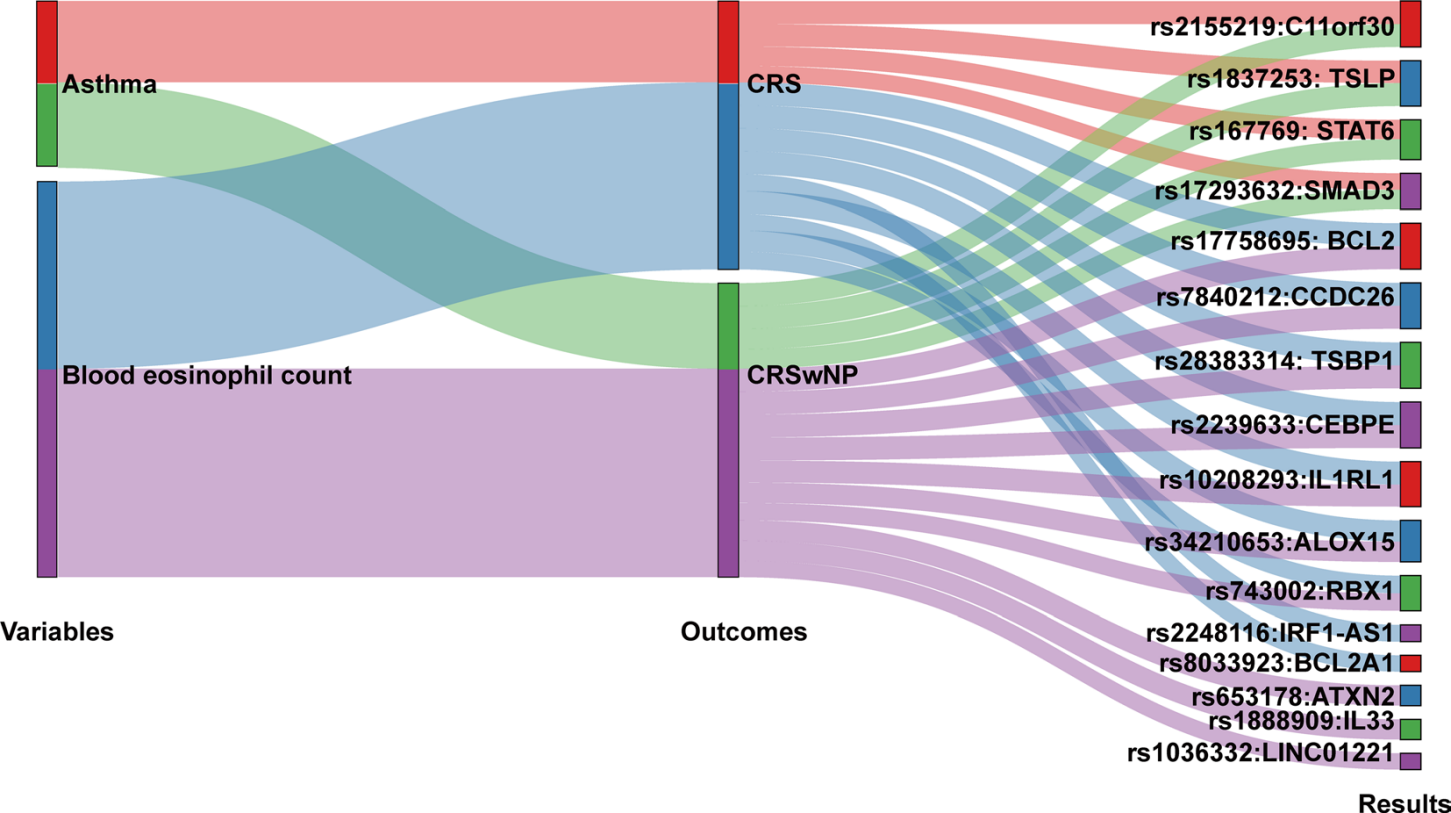
Sankey plot displays the posterior probabilities for genes with evidence of colocalization. Variables, including asthma and blood eosinophil count, were colocalized with CRS and CRSwNP. The results were shown in right part of the plot. The thickness of the curve from outcomes to results represent values of posterior probabilities. Only the results with posterior probability ≥ 0.70 are reported here (power calculations see Table S7-S10 in Additional file 2). Abbreviations: CRS, chronic rhinosinusitis; CRSwNP, chronic rhinosinusitis with nasal polyps

Finally, we performed Bayesian colocalization to identify shared genetic signals associated with blood eosinophil count, asthma, CRS and CRSwNP. This analysis identified 4 shared loci (*C11orf30*,* TSLP*,* STAT6*,* and SMAD3*) colocalized among asthma, CRS and CRSwNP, 7 shared loci (*BCL2*,* CCDC26*,* TSBP1*,* CEBPE*,* IL1RL1*,* ALOX15*,* and RBX1*) colocalized among blood eosinophil count, CRS and CRSwNP, 2 unique loci (*IRF1-AS1 and BCL2A1*) colocalized between blood eosinophil count and CRS, and 3 unique loci (*ATXN2*,* IL33*,* and LINC01221*) colocalized between blood eosinophil count and CRSwNP (Fig. 5, Table S7-S10 in Additional file 2).

## Discussion

By leveraging large GWAS datasets, our study first provided insights into the shared genetic architecture underlying asthma, blood eosinophils levels, and CRS. These shared genes may be prioritized indicators for treatment in CRS patients with multiple comorbidities from genetic view.

We found blood eosinophil count and asthma were two independent causal factors for CRS and CRSwNP in MR analysis. Mechanism research showed that activated eosinophils release abundant eosinophilic granule proteins, cytokines, chemokines, and lipid mediators that participate in epithelial damage, new bone formation and olfactory dysfunction in CRSwNP patients [27–31]. Preclinical studies also suggested that patients with higher baseline blood eosinophil counts had greater efficacy in mepolizumab treatment for CRSwNP [32, 33]. In this regard, we further emphasized the causal role of blood eosinophils in CRS or CRSwNP pathogenesis from genetic view. We also found asthma exerted causal effect on CRS or CRSwNP in our study. A strong association between asthma and CRS (OR = 3.47) was primarily observed in large sample of epidemiological survey in Europe [34]. Bronchial biopsies of asthmatic children show marked remodeling very early that may even predate the onset of nasal symptoms and may occur in absence of eosinophilic inflammation [35, 36]. Comparing nasal mucosa, which is more highly adapted to meet environment insults, lower airways are more susceptible [37]. Hence, these early abnormalities from asthma patients in lower airways may increase CRSwNP susceptibility in later life.

Currently, the “one airway, one disease” concept that upper and lower airway disorders exhibit similar pathophysiological or inflammatory profiles has been well established [37, 38], which lay a theoretical foundation for disease prevention and treatment especially when a single CRS patient has multiple comorbid manifestations of type 2 inflammation. Here, using colocalization analysis, 4 shared genes (*C11orf30*, *TSLP*, *STAT6*, and *SMAD3*) between asthma and CRS, 12 shared loci (*BCL2*,* CCDC26*,* TSBP1*,* CEBPE*,* IL1RL1*,* ALOX15*,* RBX1*,* IRF-AS1*,* BCL2A1*,* ATXN2*,* IL33* and *LIN01221*) between blood eosinophil count and CRS were colocalized. Among these genes, target for *TSLP* is licensed for severe asthma and in the clinical phase for CRSwNP [39]. Other colocalized genes (*STAT6*,* SMAD3*,* BCL2*,* CEBPE*,* IL1RL1*,* ALOX15*) have been validated well in mechanism studies in CRSwNP [40–45], which may be potential new targets for comorbidity issues of type 2 inflammation. Except for above verified genes, we found blood eosinophil count had 2 (*IRF-AS1* and *BCL2A1*) and 3 unique loci (*ATXN2*,* IL33* and *LIN01221*) for CRS and CRSwNP, respectively. Monoclonal antibody target for IL-33 has been on the stage of clinical trial in CRSwNP [46]. *BCL2A1* is mainly expressed in hematopoietic system, where it facilitates mast cell, lymphocyte and macrophage activation, thus promotes inflammation response [47]. Ataxin-2, which is encoded by the *ATXN2* gene, is a multifunctional protein of the rough endoplasmic reticulum, where it modulates mTOR signals, which has played an important role in CRSwNP pathogenesis [48]. However, the gene function of *IRF-AS1* and *LIN01221* in inflammatory process have not been reported yet.

However, there are also several limitations in our study. Firstly, due to the lack of GWAS summary statistics in Chinese populations, the conclusions of the MR study were primarily based on European ancestry populations, especially FinnGen data have a well-established genetic isolate and a unique gene pool distinguished from other Europeans [49], which may have population-specific genetic variations affecting generalizability. Secondly, we only included CRS patients from a single center in the retrospective study. There may be some selection bias. Thirdly, the number of CRS and CRSwNP cases remains relatively small. SNPs extracted from the CRS or CRSwNP were limited and highly overlapped with asthma and blood eosinophil count. Thus, reverse causation between asthma and CRS or CRSwNP could not be achieved using MR analysis. Fourthly, although our study suggests potential therapeutic targets, the clear pathway for translation into clinical practice may need further investigations.

## Conclusions

In conclusion, the present study confirmed a potential causal link between blood eosinophil count or asthma with CRS. And we found 4 shared genes between asthma and CRS or CRSwNP, 12 shared loci between blood eosinophil count and CRS or CRSwNP. Except for these developed targets (*TSLP* and *IL33*), other genes may be potential new targets for comorbidity issues of type 2 inflammation and may prioritize for mechanism research in near future.

## Electronic supplementary material

Below is the link to the electronic supplementary material.

Supplementary Material 1

Supplementary Material 2

## Data Availability

No datasets were generated or analysed during the current study.

## References

[1] Fokkens WJ, Lund VJ, Hopkins C, Hellings PW, Kern R, Reitsma S, et al. European position paper on rhinosinusitis and nasal polyps 2020. Rhinology. 2020;58(Suppl S29):1–464. 10.4193/Rhin20.600. 32077450 10.4193/Rhin20.600

[2] Laidlaw TM, Mullol J, Woessner KM, Amin N, Mannent LP. Chronic rhinosinusitis with nasal polyps and asthma. J Allergy Clin Immunol Pract. 2021;9(3):1133–41. 10.1016/j.jaip.2020.09.063. 33065369 10.1016/j.jaip.2020.09.063

[3] Khan A, Huynh TMT, Vandeplas G, Joish VN, Mannent LP, Tomassen P, et al. The GALEN rhinosinusitis cohort: chronic rhinosinusitis with nasal polyps affects health-related quality of life. Rhinology. 2019;57(5):343–51. 10.4193/Rhin19.158. 31318362 10.4193/Rhin19.158

[4] Tomassen P, Vandeplas G, Van Zele T, Cardell LO, Arebro J, Olze H, et al. Inflammatory endotypes of chronic rhinosinusitis based on cluster analysis of biomarkers. J Allergy Clin Immunol. 2016;137(5):1449–e14561444. 10.1016/j.jaci.2015.12.1324. 26949058 10.1016/j.jaci.2015.12.1324

[5] Kristjansson RP, Benonisdottir S, Davidsson OB, Oddsson A, Tragante V, Sigurdsson JK, et al. A loss-of-function variant in ALOX15 protects against nasal polyps and chronic rhinosinusitis. Nat Genet. 2019;51(2):267–76. 10.1038/s41588-018-0314-6. 30643255 10.1038/s41588-018-0314-6

[6] Demenais F, Margaritte-Jeannin P, Barnes KC, Cookson WOC, Altmüller J, Ang W, et al. Multiancestry association study identifies new asthma risk loci that colocalize with immune-cell enhancer marks. Nat Genet. 2018;50(1):42–53. 10.1038/s41588-017-0014-7. 29273806 10.1038/s41588-017-0014-7 PMC5901974

[7] Daya M, Rafaels N, Brunetti TM, Chavan S, Levin AM, Shetty A, et al. Association study in African-admixed populations across the Americas recapitulates asthma risk loci in non-African populations. Nat Commun. 2019;10(1):880. 10.1038/s41467-019-08469-7. 30787307 10.1038/s41467-019-08469-7 PMC6382865

[8] Ferreira MAR, Mathur R, Vonk JM, Szwajda A, Brumpton B, Granell R, et al. Genetic architectures of Childhood- and Adult-Onset asthma are partly distinct. Am J Hum Genet. 2019;104(4):665–84. 10.1016/j.ajhg.2019.02.022. 30929738 10.1016/j.ajhg.2019.02.022 PMC6451732

[9] Pividori M, Schoettler N, Nicolae DL, Ober C, Im HK. Shared and distinct genetic risk factors for childhood-onset and adult-onset asthma: genome-wide and transcriptome-wide studies. Lancet Respir Med. 2019;7(6):509–22. 10.1016/s2213-2600(19)30055-4. 31036433 10.1016/S2213-2600(19)30055-4 PMC6534440

[10] Olafsdottir TA, Theodors F, Bjarnadottir K, Bjornsdottir US, Agustsdottir AB, Stefansson OA, et al. Eighty-eight variants highlight the role of T cell regulation and airway remodeling in asthma pathogenesis. Nat Commun. 2020;11(1):393. 10.1038/s41467-019-14144-8. 31959851 10.1038/s41467-019-14144-8 PMC6971247

[11] Lal D, Brar T, Ramkumar SP, Li J, Kato A, Zhang L. Genetics and epigenetics of chronic rhinosinusitis. J Allergy Clin Immunol. 2023;151(4):848–68. 10.1016/j.jaci.2023.01.004. 36797169 10.1016/j.jaci.2023.01.004

[12] Staudacher AG, Peters AT, Carter RG, Welch KC, Stevens WW. Decreased nasal polyp eosinophils but increased mast cells in a patient with aspirin-exacerbated respiratory disease treated with Reslizumab. Ann Allergy Asthma Immunol. 2020;125(4):490–e493492. 10.1016/j.anai.2020.06.043. 32629015 10.1016/j.anai.2020.06.043 PMC7529928

[13] Laidlaw TM, Prussin C, Panettieri RA, Lee S, Ferguson BJ, Adappa ND, et al. Dexpramipexole depletes blood and tissue eosinophils in nasal polyps with no change in polyp size. Laryngoscope. 2019;129(2):E61–6. 10.1002/lary.27564. 30284267 10.1002/lary.27564

[14] Bachert C, Han JK, Desrosiers M, Hellings PW, Amin N, Lee SE, et al. Efficacy and safety of dupilumab in patients with severe chronic rhinosinusitis with nasal polyps (LIBERTY NP SINUS-24 and LIBERTY NP SINUS-52): results from two multicentre, randomised, double-blind, placebo-controlled, parallel-group phase 3 trials. Lancet. 2019;394(10209):1638–50. 10.1016/s0140-6736(19)31881-1. 31543428 10.1016/S0140-6736(19)31881-1

[15] Frohlich M, Olivenstein R, Cormier M. Eosinophilic pulmonary complications of dupilumab in 2 patients with asthma and chronic rhinosinusitis with nasal polyps. J Allergy Clin Immunol Pract. 2022;10(2):617–9. 10.1016/j.jaip.2021.11.029. 34929373 10.1016/j.jaip.2021.11.029

[16] Castro M, Corren J, Pavord ID, Maspero J, Wenzel S, Rabe KF, et al. Dupilumab efficacy and safety in Moderate-to-Severe uncontrolled asthma. N Engl J Med. 2018;378(26):2486–96. 10.1056/NEJMoa1804092. 29782217 10.1056/NEJMoa1804092

[17] Fokkens WJ, Lund VJ, Mullol J, Bachert C, Alobid I, Baroody F, et al. EPOS 2012: European position paper on rhinosinusitis and nasal polyps 2012. A summary for otorhinolaryngologists. Rhinology. 2012;50(1):1–12. 10.4193/Rhino12.000. 22469599 10.4193/Rhino12.000

[18] Chen C, Lu FC. The guidelines for prevention and control of overweight and obesity in Chinese adults. Biomed Environ Sci. 2004; 17 Suppl (1–36). 15807475

[19] Bulik-Sullivan BK, Loh PR, Finucane HK, Ripke S, Yang J, Patterson N, et al. LD score regression distinguishes confounding from polygenicity in genome-wide association studies. Nat Genet. 2015;47(3):291–5. 10.1038/ng.3211. 25642630 10.1038/ng.3211 PMC4495769

[20] Hartwig FP, Borges MC, Horta BL, Bowden J, Davey Smith G. Inflammatory biomarkers and risk of schizophrenia: A 2-Sample Mendelian randomization study. JAMA Psychiatry. 2017;74(12):1226–33. 10.1001/jamapsychiatry.2017.3191. 29094161 10.1001/jamapsychiatry.2017.3191 PMC6583386

[21] Bowden J, Davey Smith G, Haycock PC, Burgess S. Consistent Estimation in Mendelian randomization with some invalid instruments using a weighted median estimator. Genet Epidemiol. 2016;40(4):304–14. 10.1002/gepi.21965. 27061298 10.1002/gepi.21965 PMC4849733

[22] Hemani G, Bowden J, Davey Smith G. Evaluating the potential role of Pleiotropy in Mendelian randomization studies. Hum Mol Genet. 2018;27(R2):R195–208. 10.1093/hmg/ddy163. 29771313 10.1093/hmg/ddy163 PMC6061876

[23] Verbanck M, Chen CY, Neale B, Do R. Detection of widespread horizontal Pleiotropy in causal relationships inferred from Mendelian randomization between complex traits and diseases. Nat Genet. 2018;50(5):693–8. 10.1038/s41588-018-0099-7. 29686387 10.1038/s41588-018-0099-7 PMC6083837

[24] Giambartolomei C, Vukcevic D, Schadt EE, Franke L, Hingorani AD, Wallace C, Plagnol V. Bayesian test for colocalisation between pairs of genetic association studies using summary statistics. PLoS Genet. 2014;10(5):e1004383. 10.1371/journal.pgen.1004383. 24830394 10.1371/journal.pgen.1004383 PMC4022491

[25] Levin MG, Tsao NL, Singhal P, Liu C, Vy HMT, Paranjpe I, et al. Genome-wide association and multi-trait analyses characterize the common genetic architecture of heart failure. Nat Commun. 2022;13(1):6914. 10.1038/s41467-022-34216-6. 36376295 10.1038/s41467-022-34216-6 PMC9663424

[26] Gong W, Guo P, Li Y, Liu L, Yan R, Liu S, et al. Role of the Gut-Brain Axis in the shared genetic etiology between Gastrointestinal tract diseases and psychiatric disorders: A Genome-Wide pleiotropic analysis. JAMA Psychiatry. 2023;80(4):360–70. 10.1001/jamapsychiatry.2022.4974. 36753304 10.1001/jamapsychiatry.2022.4974 PMC9909581

[27] Jo S, Lee SH, Jo HR, Weon S, Jeon C, Park MK, et al. Eosinophil-derived TGFβ1 controls the new bone formation in chronic rhinosinusitis with nasal polyps. Rhinology. 2023;61(4):338–47. 10.4193/Rhin22.439. 37083114 10.4193/Rhin22.439

[28] Ponikau JU, Sherris DA, Kephart GM, Kern EB, Congdon DJ, Adolphson CR, et al. Striking deposition of toxic eosinophil major basic protein in mucus: implications for chronic rhinosinusitis. J Allergy Clin Immunol. 2005;116(2):362–9. 10.1016/j.jaci.2005.03.049. 16083791 10.1016/j.jaci.2005.03.049

[29] Saitoh T, Kusunoli T, Yao T, Kawano K, Kojima Y, Miyahara K, et al. Relationship between epithelial damage or basement membrane thickness and eosinophilic infiltration in nasal polyps with chronic rhinosinusitis. Rhinology. 2009;47(3):275–9. 10.4193/Rhin08.109. 19839250 10.4193/Rhin08.109

[30] Tsuda T, Maeda Y, Nishide M, Koyama S, Hayama Y, Nojima S, et al. Eosinophil-derived neurotoxin enhances airway remodeling in eosinophilic chronic rhinosinusitis and correlates with disease severity. Int Immunol. 2019;31(1):33–40. 10.1093/intimm/dxy061. 30239772 10.1093/intimm/dxy061 PMC6364622

[31] Wu D, Liu Z, Bleier BS, Huang X, Hong J. Olfactory cleft mucus eosinophil-derived neurotoxin better reflects olfactory loss than blood eosinophil counts in patients with chronic rhinosinusitis. Int Forum Allergy Rhinol. 2023. 10.1002/alr.23202. 37264735 10.1002/alr.23202

[32] Bachert C, Sousa AR, Han JK, Schlosser RJ, Sowerby LJ, Hopkins C, et al. Mepolizumab for chronic rhinosinusitis with nasal polyps: treatment efficacy by comorbidity and blood eosinophil count. J Allergy Clin Immunol. 2022;149(5):1711–e17211716. 10.1016/j.jaci.2021.10.040. 35007624 10.1016/j.jaci.2021.10.040

[33] Han JK, Bachert C, Fokkens W, Desrosiers M, Wagenmann M, Lee SE, et al. Mepolizumab for chronic rhinosinusitis with nasal polyps (SYNAPSE): a randomised, double-blind, placebo-controlled, phase 3 trial. Lancet Respir Med. 2021;9(10):1141–53. 10.1016/s2213-2600(21)00097-7. 33872587 10.1016/S2213-2600(21)00097-7

[34] Jarvis D, Newson R, Lotvall J, Hastan D, Tomassen P, Keil T, et al. Asthma in adults and its association with chronic rhinosinusitis: the GA2LEN survey in Europe. Allergy. 2012;67(1):91–8. 10.1111/j.1398-9995.2011.02709.x. 22050239 10.1111/j.1398-9995.2011.02709.x

[35] Kariyawasam HH, Aizen M, Barkans J, Robinson DS, Kay AB. Remodeling and airway hyperresponsiveness but not cellular inflammation persist after allergen challenge in asthma. Am J Respir Crit Care Med. 2007;175(9):896–904. 10.1164/rccm.200609-1260OC. 17272787 10.1164/rccm.200609-1260OC

[36] Pohunek P, Warner JO, Turzíková J, Kudrmann J, Roche WR. Markers of eosinophilic inflammation and tissue re-modelling in children before clinically diagnosed bronchial asthma. Pediatr Allergy Immunol. 2005;16(1):43–51. 10.1111/j.1399-3038.2005.00239.x. 15693911 10.1111/j.1399-3038.2005.00239.x

[37] Samitas K, Carter A, Kariyawasam HH, Xanthou G. Upper and lower airway remodelling mechanisms in asthma, allergic rhinitis and chronic rhinosinusitis: the one airway concept revisited. Allergy. 2018;73(5):993–1002. 10.1111/all.13373. 29197105 10.1111/all.13373

[38] Niimi A. Redefining one airway, one disease: broader classification considering specific pathophysiology and treatment. Respir Investig. 2021;59(5):573–5. 10.1016/j.resinv.2021.04.008. 34127424 10.1016/j.resinv.2021.04.008

[39] Hoy SM, Tezepelumab. First Approval Drugs. 2022;82(4):461–8. 10.1007/s40265-022-01679-2. 35184265 10.1007/s40265-022-01679-2

[40] Chen J, Chen S, Gong G, Yang F, Chen J, Wang Y. Inhibition of IL-4/STAT6/IRF4 signaling reduces the epithelial-mesenchymal transition in eosinophilic chronic rhinosinusitis with nasal polyps. Int Immunopharmacol. 2023;121(110554). 10.1016/j.intimp.2023.110554. 10.1016/j.intimp.2023.110554 37385124

[41] Wang M, Ye T, Liang N, Huang Z, Cui S, Li Y, et al. Differing roles for TGF-β/Smad signaling in osteitis in chronic rhinosinusitis with and without nasal polyps. Am J Rhinol Allergy. 2015;29(5):e152–159. 10.2500/ajra.2015.29.4241. 26265084 10.2500/ajra.2015.29.4241

[42] Castano R, Bossé Y, Endam LM, Desrosiers M. Evidence of association of interleukin-1 receptor-like 1 gene polymorphisms with chronic rhinosinusitis. Am J Rhinol Allergy. 2009;23(4):377–84. 10.2500/ajra.2009.23.3303. 19671251 10.2500/ajra.2009.23.3303

[43] Yan B, Wang Y, Li Y, Wang C, Zhang L. Inhibition of arachidonate 15-lipoxygenase reduces the epithelial-mesenchymal transition in eosinophilic chronic rhinosinusitis with nasal polyps. Int Forum Allergy Rhinol. 2019;9(3):270–80. 10.1002/alr.22243. 30452122 10.1002/alr.22243

[44] Scapa VI, Ramakrishnan VR, Kingdom TT. Upregulation of Bcl-2 in nasal polyps from patients with cystic fibrosis. Int Forum Allergy Rhinol. 2013;3(3):199–203. 10.1002/alr.21107. 23135964 10.1002/alr.21107 PMC7159725

[45] Cho JS, Kim JA, Park JH, Park IH, Han IH, Lee HM. Toll-like receptor 4-mediated expression of interleukin-32 via the c-Jun N-terminal kinase/protein kinase B/cyclic adenosine monophosphate response element binding protein pathway in chronic rhinosinusitis with nasal polyps. Int Forum Allergy Rhinol. 2016;6(10):1020–8. 10.1002/alr.21792. 27173130 10.1002/alr.21792

[46] Hong H, Liao S, Chen F, Yang Q, Wang DY. Role of IL-25, IL-33, and TSLP in triggering united airway diseases toward type 2 inflammation. Allergy. 2020;75(11):2794–804. 10.1111/all.14526. 32737888 10.1111/all.14526

[47] Vogler M. BCL2A1: the underdog in the BCL2 family. Cell Death Differ. 2012;19(1):67–74. 10.1038/cdd.2011.158. 22075983 10.1038/cdd.2011.158 PMC3252829

[48] Zhong B, Du J, Liu F, Liu Y, Liu S, Xie L, et al. Activation of the mTOR/HIF-1α/VEGF axis promotes M1 macrophage polarization in non-eosinophilic chronic rhinosinusitis with nasal polyps. Allergy. 2022;77(2):643–6. 10.1111/all.15050. 34390596 10.1111/all.15050

[49] Peltonen L, Jalanko A, Varilo T. Molecular genetics of the Finnish disease heritage. Hum Mol Genet. 1999;8(10):1913–23. 10.1093/hmg/8.10.1913. 10469845 10.1093/hmg/8.10.1913

