# Supplementary material for: Shared genetic investigation of asthma and blood eosinophils in relation to chronic rhinosinusitis
Source: Allergy Asthma Clin Immunol. 2025 Mar 17;21:11. doi: 10.1186/s13223-025-00956-5 (PMC11912634; doi:10.1186/s13223-025-00956-5)
Supplement: Supplementary file 1 — Supplementary Material 1 [file 13223_2025_956_MOESM1_ESM.docx]

**Datasets used in the genetic study part**

***Asthma data sets***

The datasets for asthma were from the 66 GWAS studies, of which 56 included people with European ancestry (19,954 cases, 107,715 controls), seven involved people with African ancestry (2,149 cases, 6,055 controls), two were Japanese studies (1,239 cases, 3,976 controls), and one study focused on Latino people (606 cases, 792 controls). To generate larger sample sizes for GWAS meta-analysis of asthma enabling the discovery of common novel risk loci, investigators with genome-wide data available in >142,000 individuals of diverse ancestries formed the Trans-National Asthma Genetic Consortium (TAGC)[1]. All subjects provided informed consent to participate in genetic studies and each study protocol was approved by local ethics committees of the different institutions. The definition of asthma was based on a physician’s diagnosis and/or standardized questionnaires. Imputation, quality control (including adjustments for population stratification), and specific analysis processes were based on a previously published study [1]. The summary statistics for the meta-analysis support the findings of this study and are available through a link from the GWAS Catalog entry for the TAGC study on the website (https://www.ebi.ac.uk/gwas/downloads/summary-statistics). For the purpose of this study, we extracted the datasets for the population with European-ancestry for our MR analysis.

***White blood cell types data sets***

The datasets for the white blood cell types, including absolute counts and percentage data for the lymphocytes, neutrophils, eosinophils, and monocytes were derived from a large GWAS on 173,480 European-ancestry participants[2]. The total study samples consisted of three large-scale UK studies, with 87,265 individuals from UKBB, 45,694 individuals from UK BiLEVE (a selected subset of the UKBB cohort), and 40,251 individuals from INTERVAL. White blood cell types were measured using clinical hematology analyzers at the centralized processing laboratory UK Biocenter (Stockport, UK). Genotyping was performed on the Affymetrix Gene Titan Multi-Channel Instrument according to the Affymetrix Axiom 2.0 Assay Automated Workflow. Detailed information for participants and ethic statement, genotype imputation, quality control, and association analysis can be found in a previously published study [2]. The GWAS summary statistics for the white blood cell traits are available online (<https://gwas.mrcieu.ac.uk/>).

***Chronic rhinosinusitis and nasal polyps data sets***

Summary statistics of outcome variables were obtained from FinnGen Data Freeze 8 (phenocode=J10_CHRONSINUSITIS and J10_NASALPOLYP for chronic rhinosinusitis and CRSwNP, respectively) that consists of 342,499 Finnish individuals. The GWAS were consisted of 14,369 chronic rhinosinusitis cases, 5,554 CRSwNP cases and 258,553 controls. FinnGen is a large public-private partnership aiming to collect and analyze genome and health data from 500,000 Finnish biobank participants. Details of study design, participants and ethic statement, genotyping, imputation and quality control methods have been described in a latest study [3]. Data are available online (<https://www.finngen.fi/en>).

**Instrumental variable selection**

SNPs associated with white blood cell traits or asthma and genome-wide significance (P<8.31×10-9) [2, 4] were extracted, and clumped to obtain independent loci using a threshold of linkage disequilibrium r2>0.001 and a distance of 10,000kb [2]. SNPs were not available in the outcome dataset were dropped. The exposure and outcome data were harmonized, palindromic SNPs with intermediate allele frequencies removed, and SNPs from the major histocompatibility complex region (chr6:20,000,000–40,000,000, GRCh37), which likely possess pleiotropic effects, dropped. The F parameter was estimated to evaluate instrument strength [5], using the following formula: F= (N − K − 1)/K×R2/ (1 − R2), where N is the sample size, K is the number of SNPs, and R2 is the proportion of variance explained [6]. Typically, a threshold of F>10 is recommended when defining instrument strength in MR analysis [7].

**Supplementary References**

E1. Demenais F, Margaritte-Jeannin P, Barnes KC, Cookson WOC, Altmüller J, Ang W, et al. Multiancestry association study identifies new asthma risk loci that colocalize with immune-cell enhancer marks. Nat Genet. 2018; 50(1):42-53. doi:10.1038/s41588-017-0014-7.

E2. Astle WJ, Elding H, Jiang T, Allen D, Ruklisa D, Mann AL, et al. The Allelic Landscape of Human Blood Cell Trait Variation and Links to Common Complex Disease. Cell. 2016; 167(5):1415-1429.e1419. doi:10.1016/j.cell.2016.10.042.

E3. Kurki MI, Karjalainen J, Palta P, Sipilä TP, Kristiansson K, Donner KM, et al. FinnGen provides genetic insights from a well-phenotyped isolated population. Nature. 2023; 613(7944):508-518. doi:10.1038/s41586-022-05473-8.

E4. Siedlinski M, Jozefczuk E, Xu X, Teumer A, Evangelou E, Schnabel RB, et al. White Blood Cells and Blood Pressure: A Mendelian Randomization Study. Circulation. 2020; 141(16):1307-1317. doi:10.1161/circulationaha.119.045102.

E5. Burgess S, Davies NM, Thompson SG. Bias due to participant overlap in two-sample Mendelian randomization. Genet Epidemiol. 2016; 40(7):597-608. doi:10.1002/gepi.21998.

E6. Burgess S, Dudbridge F, Thompson SG. Combining information on multiple instrumental variables in Mendelian randomization: comparison of allele score and summarized data methods. Stat Med. 2016; 35(11):1880-1906. doi:10.1002/sim.6835.

E7. Burgess S, Butterworth A, Thompson SG. Mendelian randomization analysis with multiple genetic variants using summarized data. Genet Epidemiol. 2013; 37(7):658-665. doi:10.1002/gepi.21758.
